# Dr. D. E. Fuller

**Published:** 1915-08

**Authors:** 


**Dr. D. E. Fuller**, Buffalo 1878, died in Hastings, Mich., May 13, where he had been in practice since 1881.

